# Case Report: Mastocytosis: The Long Road to Diagnosis

**DOI:** 10.3389/fimmu.2021.635909

**Published:** 2021-02-12

**Authors:** Tiago Azenha Rama, Diana Martins, Nuno Gomes, Jorge Pinheiro, Ana Nogueira, Luís Delgado, José Luís Plácido, Alice Coimbra

**Affiliations:** ^1^Serviço de Imunoalergologia, Centro Hospitalar Universitário São João, Porto, Portugal; ^2^Serviço de Imunologia Básica e Clínica, Departamento de Patologia, Faculdade de Medicina, Universidade Do Porto, Porto, Portugal; ^3^Serviço de Anatomia Patológica, Centro Hospitalar Universitário São João, Porto, Portugal; ^4^Serviço de Dermatovenereologia, Centro Hospitalar Universitário São João, Porto, Portugal; ^5^CINTESIS - Centro de Investigação em Tecnologias e Serviços de Saúde, Faculdade de Medicina, Universidade Do Porto, Porto, Portugal

**Keywords:** mastocytosis, anaphylaxis, flushing, food allergy, diagnostics

## Abstract

Mastocytosis is a heterogeneous group of disorders characterized by expansion and accumulation of clonal mast cells. Patients mainly present with either cutaneous lesions, anaphylaxis, or both. Its low prevalence and unusual features often hinder its diagnosis for several years. We report the case of an 18-year-old male who was referred to our department with a long-standing history of atypical skin lesions, allergic rhinitis, exercise-induced bronchoconstriction and what was believed to be food-related flushing and anaphylaxis, that was later diagnosed with mastocytosis. This case illustrates the need to consider investigating for mastocytosis when recurrent anaphylaxis is present, especially in the presence of atypical skin lesions, even if normal serum basal tryptase levels and allergic sensitization are present.

## Introduction

Mastocytosis is a heterogeneous group of disorders characterized by expansion and accumulation of clonal mast cells (MC). Patients mainly present with either skin lesions, anaphylaxis or both (1). MC mediator release and associated symptoms often occur in patients both with or without cutaneous involvement (2). Presentation with cutaneous lesions is particularly common among children and predominantly onsets during the first 6 months of life (3). Low prevalence and unusual features of mastocytosis often hinder its diagnosis for several years (4).

Here, we report the case of an 18-year-old male who was referred to our department with a long-standing history of atypical skin lesions, allergic rhinitis, exercise-induced bronchoconstriction and what was believed to be food allergy related flushing and anaphylaxis.

## Case Report

An 18-year-old male was referred to a Tertiary Hospital Allergy department due to recurrent anaphylaxis that was attributed to food allergy. There was a history of recurrent wheezing since early infancy, rhinitis and skin lesions on the scalp since the age of 8 months that frequently flared and relapsed. Some of these flares were associated with flushing, angioedema and presyncope. Food allergy was initially thought to be the trigger in some of these episodes. As such, at age 3 the patient was put on a strict avoidance diet and he was prescribed an adrenaline autoinjector and glucocorticoids, on demand. At this age, he also developed skin lesions suggestive of atopic dermatitis. At age 6, the patient started experiencing oral allergy symptoms with peanut and tree nuts, and throat tightness following balloon filling. This clinical picture originated marked stress and anxiety that required Psychiatry of Childhood and Adolescence follow-up. At age 12, following ingestion of egg/egg containing food, he complained of throat constriction without any other signs and symptoms. A microarray multiple allergen component assay (ImmunoCAP ISAC, Thermo Fisher Scientific, Uppsala, Sweden) was performed, showing polysensitization to food and inhalant allergens—tree nut, peanut, olive and mugwort lipid transfer proteins (LTP), major latex allergen, kiwi thaumatin-like protein, shrimp, timothy grass pollen, groups 1 and 2 allergens of house dust mites (HDM), with a total IgE of 84 kU/L, resulting in even further dietary restrictions. He was advised not to eat apple, kiwi, and peach although no oral food challenges were performed. Also, at age 12, the patient was started on monthly anti-IgE (omalizumab) for “chronic spontaneous urticaria with angioedema” that was maintained for 2 years without any improvement. In fact, he often complained of angioedema and flushing following omalizumab administration. During endoscopic knee surgery at age 17, he reportedly presented with flushing, in spite of five previous uneventful anesthetic procedures (2 inguinal hernioplasties at 2 and 6 months of age, adenoidectomy and tonsillectomy at 9 months, appendicectomy and ankle surgery for apophysitis of the calcaneus at age 11).

At the first appointment at our department, the patient complained of spells of flushing, sometimes with angioedema and/or presyncope that occurred 3–4 times/week. At this time, he had a highly restrictive diet not eating any fresh fruits other than banana, although tolerating strawberries, mango, melon, grapes and citrus. He avoided tree nuts, peanuts, chestnuts, walnuts and despite previous tolerance, as well as apple, pear, kiwi, and olive oil, as indicated by his previous physician. He had also been advised to avoid codfish and shellfish, despite tolerating salmon, hake, sardines, sea bass, swordfish, cat-fish and shrimp. He also reported mild perennial allergic rhinitis symptoms that worsened during pollen season and exercise-induced bronchoconstriction.

**Figure 1:**
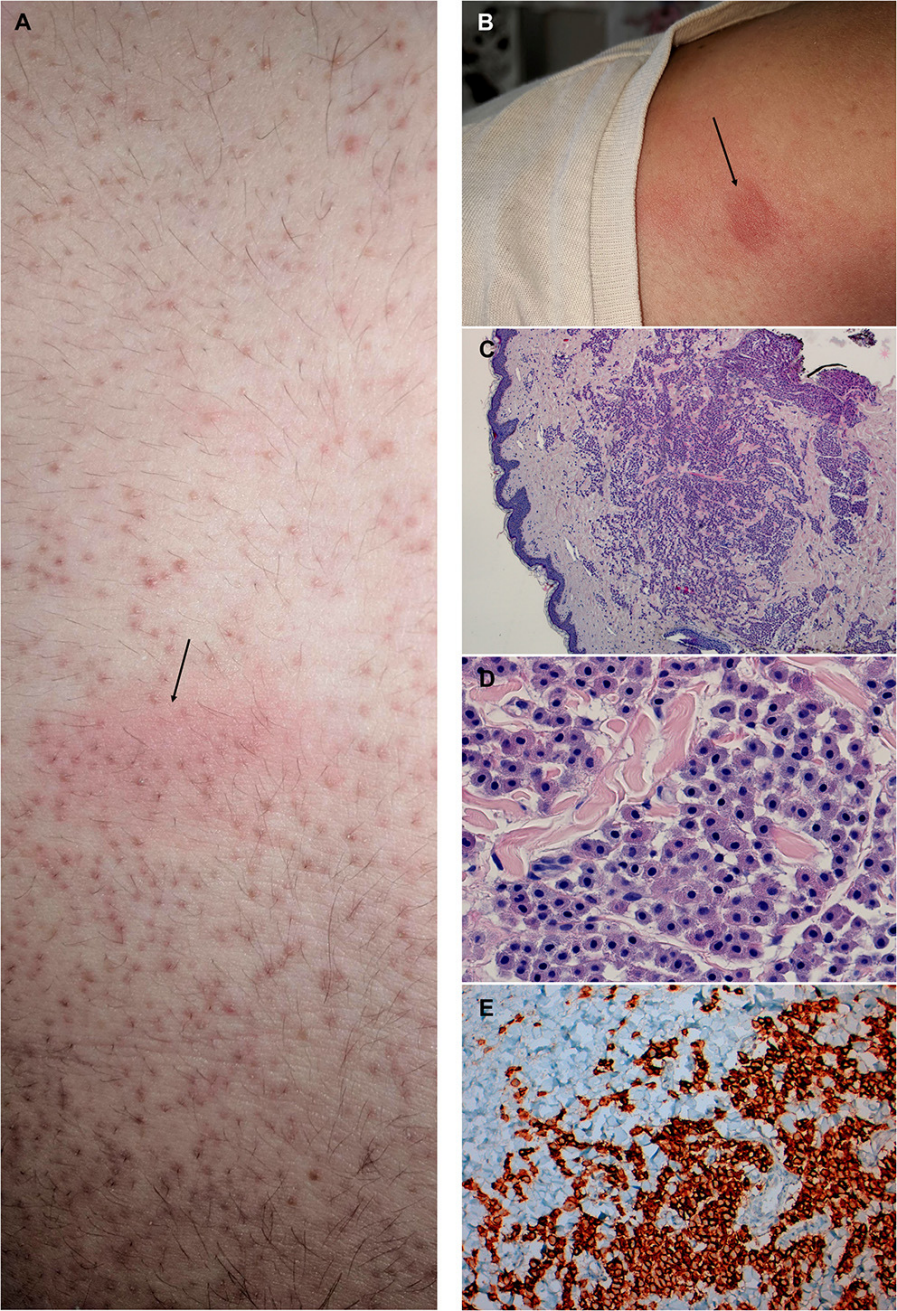
Macroscopic and histologic findings of an 18-year-old male patient with recurrent anaphylaxis, diagnosed with well-differentiated mastocytosis. **(A)** Detail of the trunk depicting exuberant widespread keratosis pilaris and a nodule (arrow) showing a wheal and flare reaction following stroking with a tongue spatula (Darier's sign). **(B)** Supraclavicular nodule showing a wheal and flare reaction following stroking. **(C)** Skin biopsy specimen of the nodule depicted in **(B)**, showing a dense nodular aggregate of mast cell in the reticular dermis, accompanied by variable numbers of interstitial mast cell (hematoxylin and eosin stain, x40 magnification); **(D)**. Mast cells with round/polygonal morphology (hematoxylin and eosin stain, x400 magnification). **(E)**. Diffuse cytoplasmatic c-kit expression in mast cells (c-kit immunohistochemistry, x100 magnification).

Physical examination was unremarkable except for the presence of exuberant widespread keratosis pilaris involving the whole trunk (Figure 1A), four scalp lesions and three nodular lesions on the trunk that developed a wheal and flare reaction upon stroking (Darier's sign, Figure 1B). Nasal and oropharyngeal inspection, cardiopulmonary auscultation and abdominal inspection were unremarkable. He was submitted to skin prick testing (SPT), with common aeroallergens, nuts, shellfish and oral allergy syndrome specific batteries (*Laboratórios LETIPharma*, Madrid, Spain), complete blood counts, specific IgE (sIgE), and serum basal tryptase measurements, and underwent oral food challenges with apple, pear, and egg. Complete blood counts did not show anemia, leukopenia or leukocytosis, or thrombocytopenia. SPT were positive for HDM and olive tree pollen, while sIgE was positive for HDM (*Dermatophagoides pteronyssinus* 51.5 kU/L, *Lepidoglyphus destructor* 32.3 kU/L), grass mix (21.8 kU/L); olive tree (4.5 kU/L); latex (11.9 kU/L), peanut (0.70 kU/L), kiwi (0.54 kU/L), and tree nut (0.40 kU/L). sIgE for both egg yolk and white, and codfish were negative. Serum basal tryptase ranged between 7 and 9.4 ng/mL, 6 months apart. Spirometry was normal with negative bronchodilation test. All oral food challenges were negative. He was referred to the dermatology department due to the exuberance of the trunk keratosis pilaris and nodules. Skin biopsy of the latter lesions showed a CD30-positive large, round/polygonal, hypergranulated mast cell infiltrate that was compatible with well-differentiated mastocytosis (WDM) (Figures 1C–E). The patient was then started on sodium cromoglycate (200 mg, 3id), rupatadine (10 mg, id), and montelukast (10 mg, id) with significant improvement in both frequency and severity of the episodes of flushing and/or angioedema. A topical nasal glucocorticoid and an on demand/pre-exertion inhaled glucocorticoid/long acting beta agonist fixed association were also prescribed. He also resumed the ingestion of egg, apple and pear with tolerance. The patient was later submitted to an abdominal ultrasound and bone densitometry which did not show any organomegalies or bone mass changes.

## Discussion

This report illustrates the case of a polysensitized young male with food allergy and a delayed diagnosis of mastocytosis, based on a skin biopsy histopathology supporting a well-differentiated mastocytosis. This case underlies the need to investigate for mastocytosis in patients with recurrent anaphylaxis, especially in the presence of longstanding skin lesions, and even with the coexistence of normal tryptase and allergic sensitization.

A recent classification categorizes mastocytosis in the skin into maculopapular cutaneous mastocytosis (CM) which also includes plaque and nodular forms, cutaneous mastocytoma and diffuse CM (5). Maculopapular CM has been subcategorized into monomorphic, formerly known as *urticaria pigmentosa*, and polymorphic variants (5). While monomorphic cutaneous involvement is easily recognized by experienced clinicians, that may not be the case for the polymorphic variants. The latter always onsets during childhood (6), presenting with lesions of varying sizes and asymmetric distribution throughout the cutaneous tegument and often involves the head and neck, namely the face and scalp, in contrast with monomorphic forms (5). Flushing is the most frequent MC mediator release related symptom (7). This form is mostly associated with low tryptase, KIT mutations involving exons other than 17 and displays distinct histopathological features, namely large, round hypergranulated MC that express CD30, having been called well-differentiated mastocytosis (7). These patients usually undergo partial or total remission during puberty/adolescence (8) but may recur during adulthood with aggressive systemic mastocytosis (SM) (9, 10). Our case displays a polymorphic maculopapular cutaneous mastocytosis, displaying a typical histopathology of WDM (hypergranulated, round MC) that underwent partial remission of skin lesions while maintaining significant MC mediator release related symptoms into adulthood.

In contrast with the general population, the most frequent causes for anaphylaxis among mastocytosis patients are Hymenoptera venom and idiopathic causes with foods triggering only 6 to 8% of anaphlaxis (11). However, among children with mastocytosis, the prevalence of food-induced anaphylaxis may reach 20% (12) to 33% (13), only surpassed by idiopathic anaphylaxis with 60% (12) to 67% (13). Our patient displayed MC mediator release symptoms that were thought to be related to food ingestion, namely egg and tree nuts and he was sensitized to LTP, thaumatin-like protein, tree nuts, peanuts, shrimp, and codfish. Oral food challenges were negative for fresh fruits containing LTP and egg. However, tree nuts and peanuts induced reproducible oral allergy syndrome manifestations that may be mediated by LTP. This case illustrates that oral food challenges are not only safe but indicated in mastocytosis patients, with suspected food allergy.

Even when partial remission occurs, persistence of mastocytosis in the skin and MC mediator release-related symptoms into adulthood mandates a bone marrow (BM) study, in order to assess systemic involvement (14). Proposed criteria for the diagnosis of well-differentiated systemic mastocytosis (WDSM) include one major criterion that is shared with the remaining forms of SM (i.e., MC aggregates in the bone marrow) and four minor criteria—expression of CD30 and/or overexpression of carboxypeptidase A through flow cytometry; clustering of BM MCs outside BM particles forming groups of ≥ 2 MC; presence of mutations involving any codon of KIT or a clonal human androgen receptor assay (HUMARA) pattern; association of female sex with either pediatric disease onset or familial aggregation in adults (7). Diagnosis of WDSM is established in the presence of the major criterion and one minor, or three minor criteria (7). As such, BM flow cytometric evaluation should include assessment of BM MC expression of, at least, CD25/CD2 (generally not present in WDSM), CD30 and carboxypeptidase A (7, 15). KIT mutational analysis should be performed, starting with D816V KIT mutation, followed by KIT sequencing, if negative (16). If both D816V and sequencing are negative, clonality should be assessed through HUMARA (7). In this case, BM study is planned, although it has not yet been performed due to constraints related to the COVID19 pandemic. The term mastocytosis in the skin has been used to differentiate patients with skin biopsy compatible with mastocytosis that have not undergone BM studies (17). Our patient has morphological and immunophenotipical features that are compatible with well-differentiated cutaneous mastocytosis (7). As such, the patient may be provisionally classified as having a well-differentiated mastocytosis in the skin.

Literature on the efficacy of omalizumab in mastocytosis is still scarce (18) especially when it comes to cutaneous mastocytosis in children (19). This monoclonal antibody binds free IgE, preventing linkage and subsequent activation of its high affinity receptor (FcεRI) expressed by MC and basophils (20). Moreover, it seems to downregulate the expression of FcεRI receptors which are thought to be also involved in MC survival and non-immune activation (21). While it has been shown to be effective in the prevention of MC mediator release symptoms in both mastocytosis (19, 22–25) and food allergy (26, 27), safety concerns have been raised by the Food and Drug Administration (FDA) following reports of anaphylaxis caused by omalizumab, later sustained by several studies (28). Prevalence of such reactions was shown to reach 0.2% among patients with asthma treated with this monoclonal antibody (29). Allergy to its excipient, polysorbate (30) and activation of MC FcγRI by IgE-omalizumab immune complexes (31) have been proposed as potential causes for such reactions. While its efficacy seems to be promising in adults (18), reports of allergic reactions among mastocytosis patients, such as this case, are scarce.

Significant delays in the diagnosis of mastocytosis are infrequently reported. However, delays may be prominent in adults, averaging 6.5 to 8.1 years (4, 32) but reaching up to 50 years (4). Years lost to diagnosis are particularly important in cases without cutaneous involvement, especially in those suffering from an aggressive form of the disease (33). Nonetheless, this may also occur in patients presenting with skin lesions and it may severely impair patients' quality of life or carry more dire consequences, such as organ dysfunction and death (in advanced forms), life-threatening anaphylaxis, or pathological bone fractures from severe osteoporosis (4, 34). Delays in diagnosis may result from the use of insensitive techniques (34), a misguided belief that normal basal tryptase values preclude mastocytosis (35), or may be due to atypical symptoms (32). While skin lesions were atypical in this case, frequent spells of flushing, angioedema and presyncope should have raised the suspicion sooner, even in face of normal basal tryptase values and significant allergic disease.

## Conclusions

This case underlines the relevance of considering the diagnosis of mastocytosis in the presence of recurrent anaphylaxis, while stressing the need of a thorough skin examination for mastocytosis skin lesions.

## Data Availability Statement

The original contributions generated for the study are included in the article/supplementary material, further inquiries can be directed to the corresponding author/s.

## Ethics Statement

Written informed consent was obtained from the individual(s) for the publication of any potentially identifiable images or data included in this article.

## Author Contributions

TR conceived and designed the case report, contributed to the clinical and pathology diagnosis, collected all data and wrote the manuscript. DM and JP contributed to the pathology diagnosis, immunohistochemistry, and its photographic material. NG and AN contributed to the dermatologic evaluation and skin biopsies. LD supervised the conception, analysis, design of the work and manuscript drafting. JP and AC consulted the patient and supervised oral food challenges. All authors critically revised the manuscript for important intellectual content, provided approval of the final version and agreed to be accountable for all aspects of the work, ensuring that questions related to the accuracy or integrity of all parts of the work are appropriately investigated and resolved.

## Conflict of Interest

The authors declare that the research was conducted in the absence of any commercial or financial relationships that could be construed as a potential conflict of interest.
